# Identification and characterization of oligomeric proanthocyanidins with significant anti-cancer activity in adzuki beans (*Vigna angularis*)

**DOI:** 10.1016/j.heliyon.2019.e02610

**Published:** 2019-10-23

**Authors:** Sei-ichi Kawahara, Chisato Ishihara, Kiriko Matsumoto, Shogo Senga, Koichiro Kawaguchi, Ayaka Yamamoto, Jutalak Suwannachot, Yasunori Hamauzu, Hidefumi Makabe, Hiroshi Fujii

**Affiliations:** a Interdisciplinary Graduate School of Science and Technology, Shinshu University, 8304 Minami-minowa Kami-ina, Nagano, 399-4598, Japan; b Graduate School of Science and Technology, Department of Biomedical Engineering, Shinshu University, 8304 Minami-minowa Kami-ina, Nagano, 399-4598, Japan; c Department of Bioscience and Biotechnology, Faculty of Agriculture, Shinshu University, 8304 Minami-minowa Kami-ina, Nagano, 399-4598, Japan; d Graduate School of Science and Technology, Department of Agriculture, Division of Food Science and Biotechnology, Shinshu University, 8304 Minami-minowa Kami-ina, Nagano, 399-4598, Japan; e Department of Biomolecular Innovation, Institute for Biomedical Sciences, Interdisciplinary Cluster for Cutting Edge Research, Shinshu University, Minami-minowa, Kami-ina, Nagano, 399-4598, Japan

**Keywords:** Analytical chemistry, Food composition, Food chemistry, Biochemistry, Cancer research, *Vigna angularis*, Proanthocyanidin, Anti-cancer, Fatty acid-binding protein

## Abstract

The aim of the present study was to characterize and evaluate the anti-cancer activity of proanthocyanidin-enriched fractions from adzuki beans. For this purpose, we concentrated proanthocyanidins from adzuki beans (*Vigna angularis*) into five fractions using Amberlite XAD-1180N, Toyopearl HW40F, and Sepacore C-18 reverse-phase flash column chromatography. Proanthocyanidin-enriched fractions were characterized as (epi)catechin hexamer, heptamer, and octamer, epigallocatechin-(epi)catechin pentamer, and epigallocatechin-(epi)catechin hexamer using electrospray ionization time-of-flight mass spectrometry and thiolytic degradation. These fractions showed significant anti-cancer activity against the human PC-3 prostate cancer cell line. They also significantly suppressed the expression of the fatty acid-binding protein 5 gene, which plays critical roles in cell growth and metastasis in prostate cancer.

## 1 Introduction

Adzuki beans (*Vigna angularis*) are an important legume that are used in traditional foods such as confectionaries (e.g., Wagashi, Manju, and Yokan) in Japan. They are cultivated in Japan and other East Asian countries. Red adzuki beans have strong antioxidant activity, and have been used in traditional Chinese medicines for various purposes [1]. The main pigments in adzuki beans are proanthocyanidins [1, 2]. Proanthocyanidins are a class of oligomeric or polymeric phenol compounds that contain flavan-3-ol units. They show a wide variety of biological activities such as suppression of endothelin 1 synthesis [3], anti-inflammatory activity [4], and inhibition of cancer cell proliferation [5, 6, 7, 8, 9, 10]. Recently, we reported that epicatechin pentamer and hexamer synthesized in our laboratory inhibited cancer metastasis and invasion [11]. The structure of proanthocyanidin consists of a catechin and/or epicatechin polymer linked through 4–8 or 4–6 interflavan bonds. Sometimes these compounds contain epigallocatechin and/or gallate. These compounds showed significant cytotoxic activity against the human PC-3 prostate cancer cell line. Moreover, they suppressed the expression of the cancer-promoting gene, fatty acid-binding protein 5 (*FABP5*) [12,13].

Proanthocyanidin-enriched fractions from with significant anti-cancer activity in adzuki bean have not been so far concentrated and characterized. Therefore, the aim of this work was to characterize and evaluate the anti-cancer activity of proanthocyanidin-enriched fractions from adzuki beans. For these purposes, we concentrated proanthocyanidins using gel filtration chromatography and analyses by high-performance liquid chromatography (HPLC).

## 2 Materials and methods

### 2.1 Materials

Amberlite XAD-1180N was purchased from Organo Corporation (Tokyo, Japan), and Toyopearl HW 40F from Tosoh Corporation (Tokyo, Japan). Adzuki beans were purchased from Imuraya Co. Ltd, Japan in 2015. Standard samples of catechin tetramer and pentamer, and epicatechin dimer to hexamer were synthesized in our laboratory [11].

### 2.2 Instruments

^1^H and ^13^C nuclear magnetic resonance (NMR) spectra were recorded on a Bruker DRX 500 spectrometer at 500 MHz for ^1^H NMR and 125 MHz for ^13^C NMR. Chemical shifts (δ ppm) in the ^1^H NMR spectra are expressed relative to the signal of tetramethylsilane adjusted to 0.00 ppm, and coupling constants are expressed in hertz. Liquid chromatography-mass spectrometry (LC-MS) was performed using a Waters Xevo QTOF (quadrupole time-of-flight) mass spectrometer and an ACQUITY UPLC (ultra-performance liquid chromatography) system.

Column chromatography was carried out using Amberlite XAD-1180N, Sephadex LH-20 (GE Healthcare), and Toyopearl HW 40F. Analytical and preparative HPLC were performed using a Shimadzu LC-10AD system with a reverse-phase HPLC column (InertSustain C18; 250 mm × 4.6 mm i.d., 3 μm particle size, GL Science, Japan).

### 2.3 Extraction of proanthocyanidins from adzuki beans

Adzuki beans (1.6 kg) were soaked in water (6 L) at room temperature for 15 h. The extracts were filtered with cotton and the solvent was concentrated in vacuo at 45 °C to yield a slurry. The slurry was applied to Amberlite XAD-1180N resin (2.0 L) and eluted with ethyl acetate (4.0 L) and methanol (4.0 L). The methanol eluate (1.8 g) was fractionated using Toyopearl HW40F (200 mL) with 20% ethanol (600 mL), 60% ethanol (700 mL), and 60% acetone (300 mL) to afford 500 mg, 200 mg, and 1.10 g of extract, respectively. The 60% acetone eluate (called as ABE: adzuki beans extracts) was further purified with flash column chromatography using a Sepacore C18 reverse-phase column (Thermo Fisher Scientific). Elution with 10%, 20%, 30%, 40% methanol in acetonitrile gave five fractions (Fr. 1–Fr. 5: 73.3 mg, 349.8 mg, 472.4 mg, 179.4 mg, and 57 mg). These samples were analyzed by LC-ESI-TOFMS.

### 2.4 HPLC method for separation of polyphenolic compounds

The crude sample was diluted in acetonitrile (10 mg/mL) and filtered through a 0.45-μm PTFE membrane filter before injection. Twenty microliters of sample were injected into the HPLC column (InertSustain C18; 250 mm × 4.6 mm i.d., 5 μm particle size) and eluted with mobile phase A (0.2% acetic acid) and phase B (acetonitrile). The flow rate was 0.2 mL/min and the column temperature was 30 °C. A gradient elution at 10 min intervals was performed as follows: 0–10 min, 100% A; 10–30 min, 94% A and 6% B; 30–45 min, 80% A and 20% B; and 45–60 min, 100% B.

### 2.5 UPLC-ESI-TOFMS analysis

LC-MS was performed using a Waters Xevo QTOF mass spectrometer equipped with an ACQUITY UPLC system. The heated capillary and spray voltage were maintained at 300 °C and 3.0 kV, respectively. Nitrogen was used at 80 psi for the sheath gas and 20 psi for the auxiliary gas. Full scan mass spectra in the range *m/z* 100–5000 were acquired in positive ion mode with a scan speed of one scan per second. The tandem mass spectrometry collision gas was helium with a collision energy of 30% of the 5 V end-cap maximum ticking voltage.

### 2.6 Thiolytic degradation of Fr. 3 in Sepacore C18 reverse-phase column chromatography and LC-MS analysis

Constitutional flavan-3-ol units of Fr. 3 were analyzed using a HPLC-DAD-ESI-MS (HPLC-diode-array-detection (DAD)-electrospray ionization (ESI)-mass spectrometry (MS)) system after thiolytic degradation, which was performed according to the method of Hamauzu et al. [14]. In a screw-cap test tube, an aliquot (100 μL) of a methanolic solution of Fr. 3 was mixed with 100 μL of methanol containing 0.30 mol/L HCl and 10% (w/v) 2-mercaptoethylamine hydrochloride (cysteamine hydrochloride). The mixture was incubated for 30 min at 75 °C. To assess the occurrence of isomerization, reaction at 50 °C for 90 min was also performed. The reaction mixture was added to two times its volume of water, filtered using a 0.45-μm membrane filter, and analyzed using an ACQUITY UPLC separation module coupled with a Waters Micromass Q-micro MS(/MS) system that was controlled with MassLynx v4.1 software (Waters, Milford, MA). The column was a Phenomenex Luna C_18_ (150 × 4.6 mm, 5 μm particle size) with a security guard cartridge (4.0 × 3.0 mm). The solvents used were: (A) 0.5% (volume fraction) formic acid in 5% (volume fraction) acetonitrile, and (B) 0.5% (volume fraction) formic acid and 20% (volume fraction) 2-propanol in acetonitrile. The gradient program started with 0% solvent B (0–1 min), and was slowly increased to 5% solvent B at 10 min, 5% B at 15 min, 40% B at 40 min, and 90% B at 50 min. The flow rate was 0.5 mL/min. The MS was performed in negative ion mode to analyze the thiolytically degraded mixture with a capillary voltage of −3.0 kV and nebulizer gas (N_2_) temperature of 350 °C (gas flow rate of 600 L/h). The cone voltage was set at 25 V. Mass spectra were scanned over the *m/z* range 100–1000. Analysis in positive ion mode was also conducted for confirmation. Thiolysis of procyanidin B2, a dimer of (−)-epicatechin was conducted to confirm the retention time of (−)-epicatechin cysteamine thioether. The peak area obtained at 280 nm was used to calculate the mean degree of polymerization (mDP) because addition of cysteamine does not affect the absorbance.

### 2.7 Cell lines, cell culture, and reagents

Human prostate cancer cell line PC-3 was purchased from the Health Science Research Resources Bank (Osaka, Japan). The cells were maintained in a monolayer culture at 37 °C and 5% CO_2_ in RPMI-1640 (R8755, Sigma-Aldrich) supplemented with 10% charcoal-stripped fetal bovine serum (No. 04-201-1, Biological Industries) and 1% antibiotic-antimycotic mixed stock solution (No. 09366-44, Nacalai Tesque). The cells were treated with various concentrations of grape stem extract for 48 h.

### 2.8 Viable cell count assay

The degree of cell proliferation was evaluated with Cell Count Reagent SF (Nacalai Tesque, No. 07553-15) according to the manufacturer's instructions. The cells were plated in 96-well plates and treated with the indicated concentrations of test compounds derived from adzuki beans extract for 48 h. The absorbance at 450 nm was measured using a microplate reader after the addition of the Cell Count Reagent.

### 2.9 Quantitative real-time PCR (qPCR)

Cells were plated in six-well plates and grown to 50% confluence. The cells were treated with the indicated concentrations of test compounds for 48 h. Total RNA was extracted from these cells using the Plant RNA Purification Reagent (Invitrogen No. 12322-012), and 1 μg of total RNA was reverse transcribed into cDNA using the ReverTra Ace quantitative real-time PCR (qPCR) RT Master Mix (Toyobo No. FSQ-301). qPCR analyses were performed with the StepOne Real-Time PCR system (Applied Biosystems/Thermo Fisher Scientific) using THUNDERBIRD SYBR qPCR Mix (Toyobo No. QPS-201).

### 2.10 Measurement of apoptosis by assay for caspase-3 activity

Assays for caspase-3 activity were carried out using BD Cytofix/Cytoperm™ Kit (BD Biosciences, No. 554714), according to the manufacture's protocol. Purified rabbit anti-active caspase-3 (BD Pharmingen™, No. 559565) was used as the first antibody (1^st^ Ab) and FITC-conjugate anti-rabbit Ig G (Jackson ImmunoResearch, No. 711-096-152) was used for the second antibody (2^nd^ Ab). Briefly, after treatment of cells with 1% w/v ABE or 500 nmol/L of carnitine palmitoyltransferase (CPT) for 48 h, the cells were collected and prepared by the same method as described in cell cycle analysis. The cells were diluted in PBS and fixed with BD Cytofix/Cytoperm Fixation and Permilization Solution on ice in the dark for 20 min. The cells were washed with the washing buffer and then reacted with 1^st^ Ab at room temperature. Next, the cells were washed with the washing buffer and then reacted with 2^nd^ Ab at room temperature. After the reaction, the cells were diluted in PBS, and flow cytometry was performed with FACSCalibur flow cytometer (Becton Dickinson, Japan), and the data obtained were analyzed utilizing Cell Quest Software. For each sample, 1 × 10^4^ cells were recorded.

### 2.11 Cell cycle analysis

Effects of test compounds on the phase distribution of cell cycle were assessed by flow cytometry. Briefly, after treatment of cells with 1% w/v ABE or the control (vehicle) for 48 h, floated cells were discarded by aspiration and the attached cells were trypsinized and thereafter washed twice with cold PBS, and centrifuged. The pellets were used for sample preparation for flow cytometry using BD Cycletest Plus DNA Reagent Kit (BD Biosciences, No.340242), according to the manufacturer's protocol. Flow cytometry was performed with FACSCalibur flow cytometer (Becton Dickinson, Japan), and the data were analyzed utilizing Cell Quest software. For each sample, 1 × 10^4^ cells were recorded.

### 2.12 Statistical analysis

GraphPad Prism 7 was used for statistical analysis. Data are expressed as means ± standard deviations. Comparisons between two groups were performed with two-tailed Student's *t*-tests. For comparisons of more than two groups, one-way analysis of variance (ANOVA) with Dunnett's post-hoc test or two-way ANOVA with Sidak's post-hoc test was used. Statistical significance is indicated by *P < 0.05 or **P < 0.01.

## 3 Results and discussion

### 3.1 Concentration of proanthocyanidins using gel filtration chromatography

**Fig. 1:**
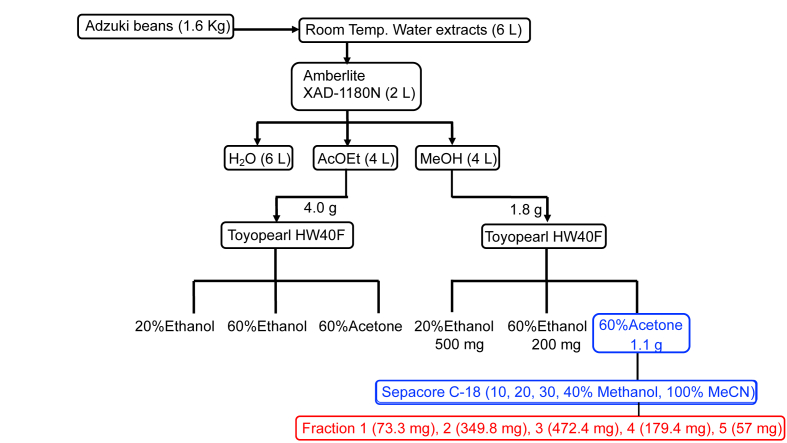
Purification procesure of water extract of adzuki beans at room temperature.

The main aims of this research were characterization and evaluation of the antitumor activity of proanthocyanidin-enriched fractions from the adzuki beans. To the best of our knowledge, this is the first study on characterization of proanthocyanidin-enriched fractions from adzuki beans, which have structures ranging from epicatechin trimer to nonamer and can contain the pyrogallol moiety. The purification scheme for the water extracts at room temperature using gel filtration chromatography is shown in Fig. 1. The use of Amberlite XAD-1180N for the first stage and Toyopearl HW 40F before HPLC purification allowed for purification of higher oligomerized proanthocyanidins.

**Fig. 2:**
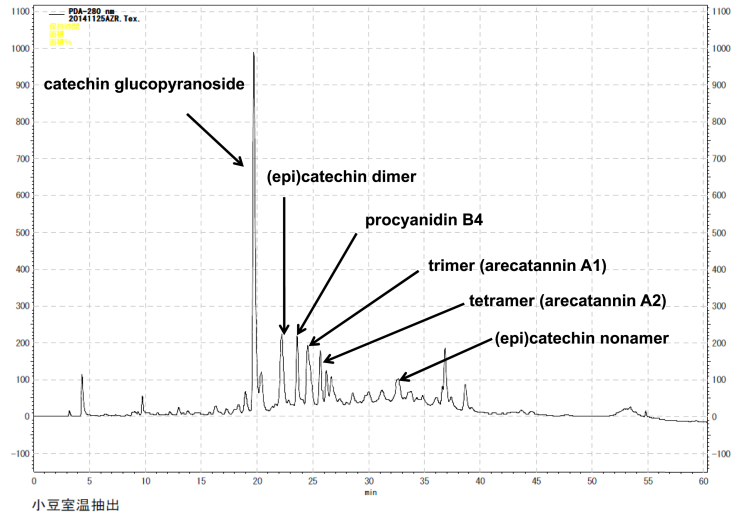
HPLC chromatographic profile of EtOAc eluted fraction using Amberlite XAD-1180N resin.

The HPLC results for the ethyl acetate-eluted fraction from the Amberlite XAD-1180N resin are shown in Fig. 2. We identified catechin glucopyranoside, (epi)catechin dimer, procyanidin B4, arecatannin A1, arecatannin A2, (epi)catechin nonamer, piceid, and dehydroquercetin rhamnoside. These compounds were identified using ESI-TOFMS, NMR, and by comparison with spectra of known compounds or synthesized procyanidin B2, B4 [15], arecatannin A1, and arecatannin A2 [11,16]. This fraction did not show significant cytotoxic activity or suppression of the expression of *FABP5*.

**Fig. 3:**
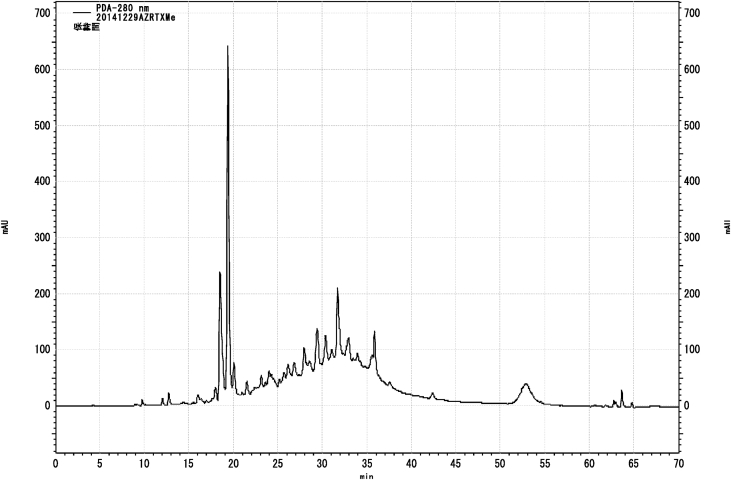
HPLC chromatogram of MeOH eluted fraction using Amerlite XAD-1180N resin.

Next, we evaluated the methanol eluted fraction from the Amberlite XAD-1180N resin because this fraction showed significant activity (Fig. 3).

**Fig. 4:**
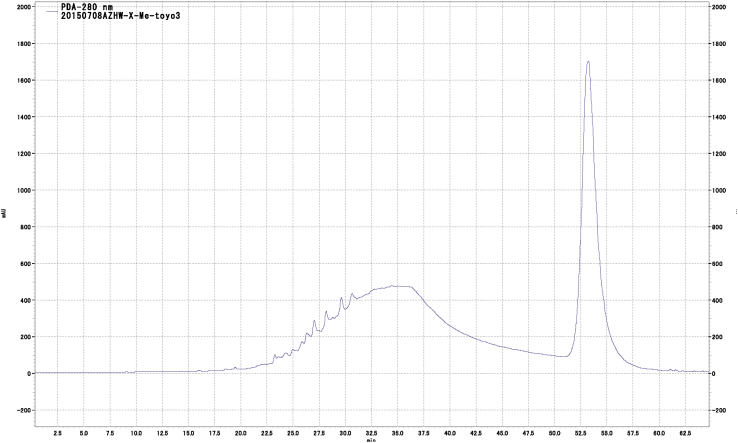
HPLC chromatogram of 60% acetone eluted fraction (ABE) using Toyopearl HW40F.

We further purified this fraction using Toyopearl HW40F to afford three fractions. Fraction 3 (60% acetone-eluted fraction from Toyopearl HW 40F, called as ABE) (Fig. 3) showed significant cytotoxic activity, and we further purified this fraction using flash chromatography with a Sepacore C18 reverse-phase column to afford five fractions (Fig. 4).

### 3.2 Thiolytic degradation products

**Fig. 5:**
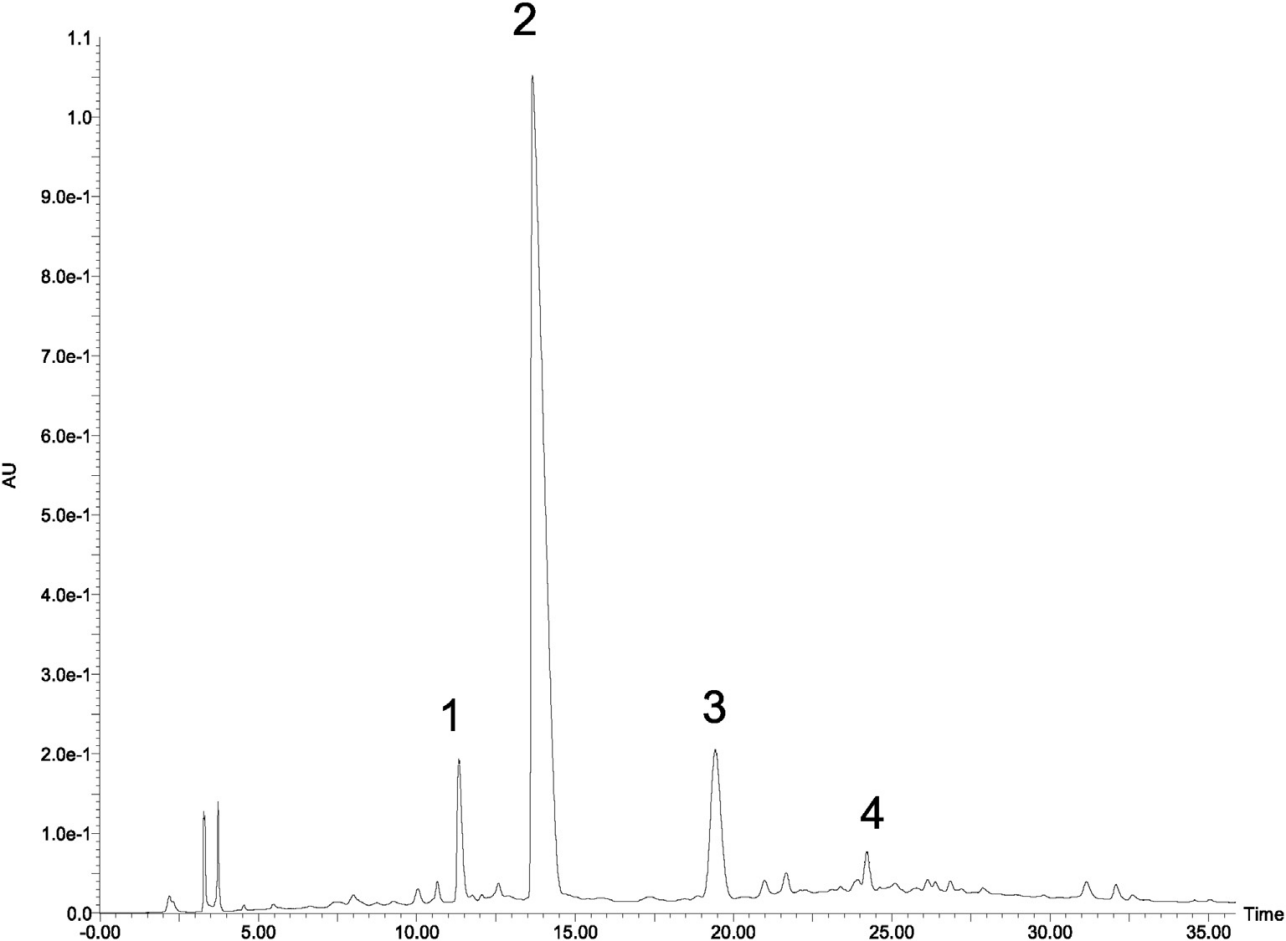
HPLC chromatogram of the thiolytic degraded products of procyanidin in Fr 3. Peaks: 1 = catechin cysteamine thioether, 2 = epicatechin cysteamine thioether, 3 = (+)-catechin, and 4 = (−)-epicatechin.

Thiolysis of Fr. 3 in the Sepacore C18 reverse-phase column revealed that the main degradation product was (−)-epicatechin cysteamine thioether (peak 2 in Fig. 1), which generated a molecular ion at *m/z* 364 ([M – H]^−^) and gave a fragment ion at *m/z* 287 in negative mode. This characteristic fragmentation of (−)-epicatechin cysteamine thioether has been reported by Selga et al. [19]. (+)-Catechin cysteamine thioether (peak 1, [M – H]^−^ at *m/z* 364) was also detected as a product corresponding to the extension unit, although it was present at a lower level than epicatechin cysteamine thioether. (+)-Catechin and (−)-epicatechin (peaks 3 and 4, respectively, [M–H]^−^ at *m/z* 289) were detected as thiolysis products without cysteamine conjugation, indicating that these are terminal units of proanthocyanidins in Fr. 3. Although (+)-catechin can be produced from (−)-epicatechin by epimerization during thiolysis at 75 °C, reaction at 50 °C could not eliminate the (+)-catechin peak. The mDP calculated using the peak area of 280 nm was seven and this was consistent with the result obtained by TOF-MS analysis, which showed that the heptamer was the main component of Fr. 3. Thus, the results suggest that Fr. 3 contains procyanidins with a mDP of around seven and constituent units composed of (−)-epicatechin as the major extension units and (+)-catechin as the major terminal units (see Fig. 5).

### 3.3 ESI-TOF-MS analysis of Fr. 3 and 4

The MS results for these fractions showed pseudo-molecular ion peaks with a difference of 288 atomic mass units (amu), which corresponded to an oligomer of (epi)catechin. For example, the peaks at *m/z* 2307, 2019, and 1731 corresponded to the masses for octamer, heptamer, and hexamer (epi)catechin units. In addition, the peaks at *m/z* 2324 and 2036 corresponded to the masses for (epi)gallocatechin containing octamer and heptamer (epi)catechin units. The data from thiol analysis suggested that it is highly possible that the terminal unit is catechin. Thus, the main component of Fr. 3 is (epicatechin)_6_-catechin. Similarly, the main components of Fr. 4 are (epicatechin)_7_-catechin and its (epi)gallocatechin containing compound.

### 3.4 Effects of various concentrations of test fractions on PC-3 prostate cancer cell proliferation

The anti-cancer activities against the PC-3 prostate cancer cell line for fractions obtained using ABE or Sepacore C18 reverse phase column chromatography are shown in Fig. 7. The Sepacore C18-purified Frs. 1–5, and especially Fr. 3 and Fr. 4, showed significant cell growth inhibitory activities. The results obtained using Fr. 3 and Fr. 4 are shown in Fig. 7. The inhibitory activities of test fractions against cell proliferation has also been observed in colorectal cancer cells (HCT116) (data not shown).

**Fig. 6:**
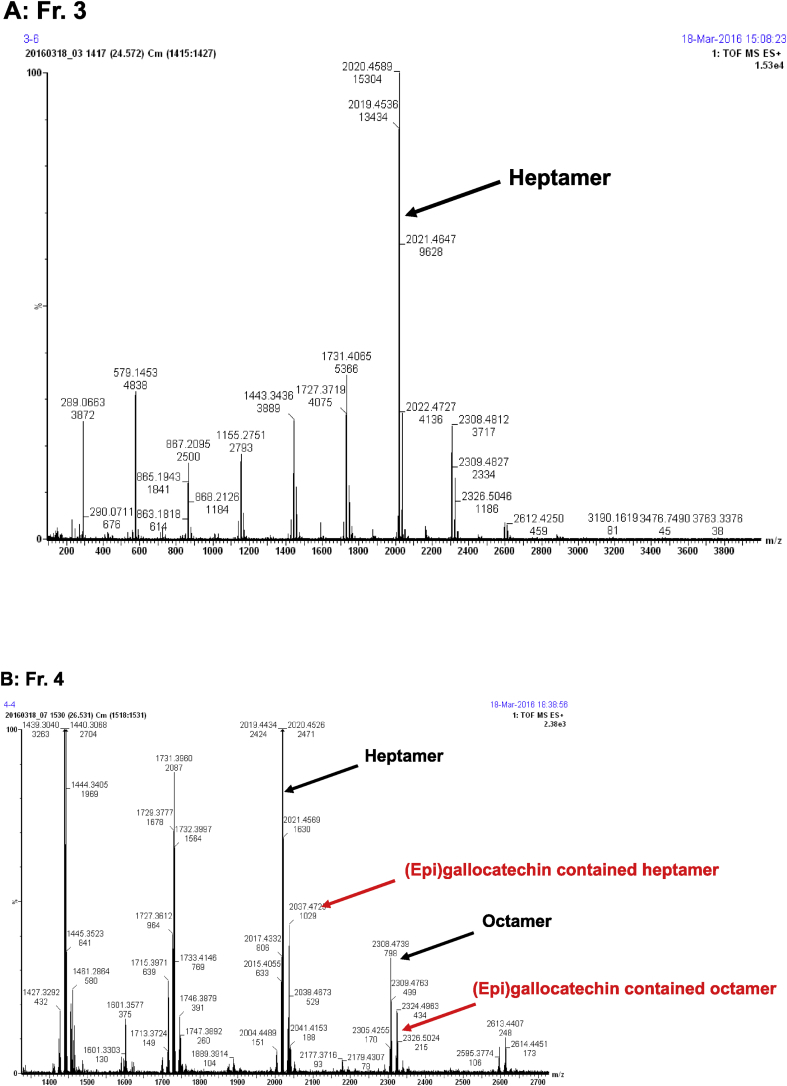
ESI-TOF-MS spectra of (A) Fr. 3 and (B) Fr. 4.

**Fig. 7:**
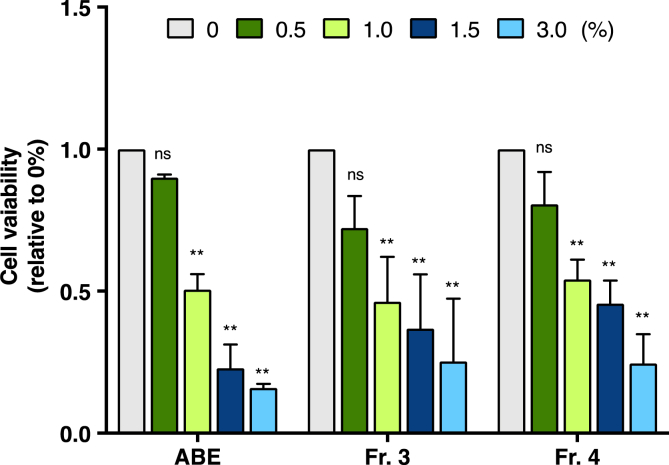
Effects of Toyopearl HW40F-purified 60% acetone fraction (ABE) or Sepacore C18-purified fractions (Fr. 3 and Fr. 4) on PC-3 prostate cancer cell proliferation. Effects of various concentrations of Sepacore C-18-purified fractions (Fr. 3 and Fr. 4) or ABE on PC-3 cancer cell proliferation. After treatment of cells with each fraction for 48 h, cell proliferation was determined by a viable cell count assay. The values presented are the rates of inhibition of cell proliferation in the treated samples compared with that in the control (vehicle). Values are expressed as the mean ± standard deviation of three independent experiments. **P < 0.01 and ns = not significant from two-way ANOVA followed by Sidak's multiple comparison test.

### 3.5 Effects of test fractions on cell cycle distribution

**Fig. 8:**
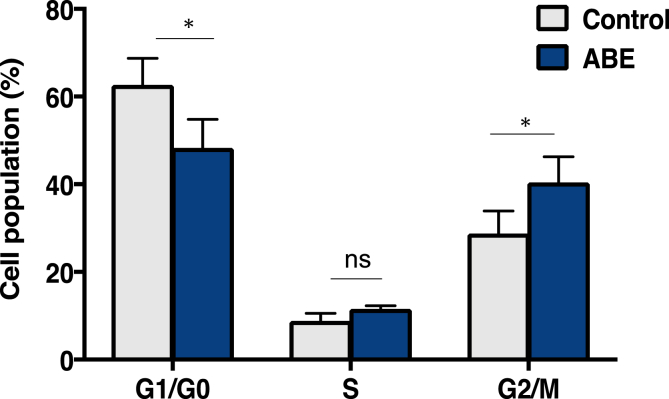
Effects of ABE on the cell cycle in PC-3 prostate cancer cells. Cells treated with the test fraction (ABE) for 48 h were collected and stained with propidium iodide using a BD Cycletest™ Plus DNA Reagent Kit (Becton Dickinson and Company BD Biosciences) obtained from Phoenix Flow Systems. Following FACS analysis, cell cycle distributions were further analyzed by Cell Quest software. *P < 0.05 and ns = not significant from two-way ANOVA followed by Sidak's multiple comparison test.

Cell growth and proliferation are related to cell cycle progression. In this study with FACS analysis, treatment of PC-3 prostate cancer cells with ABE for 48 h induced an increase in the G2/M phase population from 28.8 % to 40.5 % and a G1/G0 phase decrease from 62.7 % to 48.3 %. ABE blocked the PC-3 prostate cancer cell cycle at the G2 phase within 48 h (Fig. 8).

### 3.6 Effects of test fractions on caspase-3 activity assessed by FACS

**Fig. 9:**
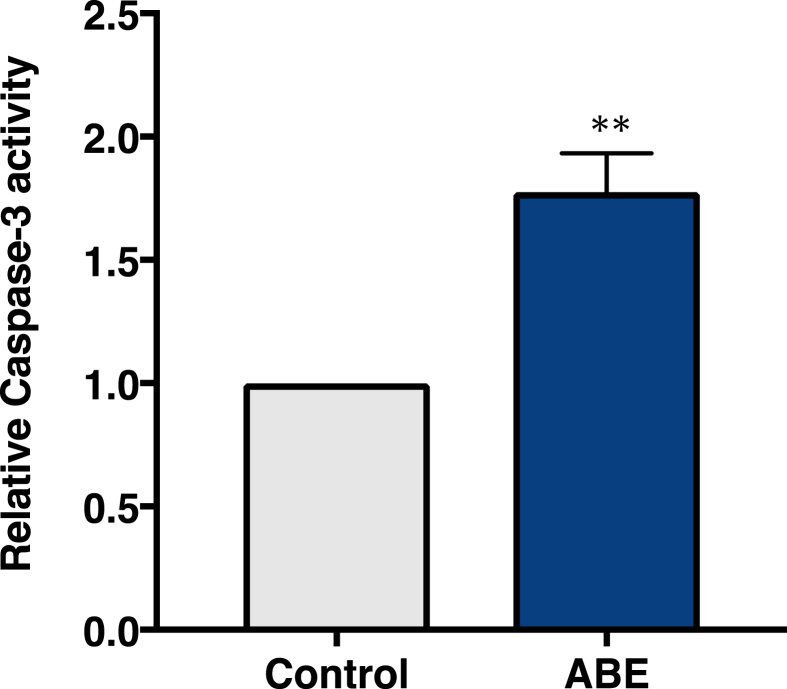
Effects of ABE on apoptosis-inducible activity in PC-3 prostate cancer cells. Five μmol/L CPT (carnitine palmitoyltransferase) or 1% w/v ABE was used for the assay. The graph presents the mean values of the FITC (fluorescein isothiocyanate) fluorescence activities. A shift of the mean FITC fluorescence activities of mock cells to the right side was taken as an increase in apoptosis. The values are presented the rate of induction of apoptosis compared with that of the control (vehicle). Data were analyzed using Student's t-test (**P < 0.01).

Next, we examined whether ABE could induce apoptosis in PC-3 cells. We evaluated the caspase-3 activity to elucidate that cells exposed to ABE were actually undergoing apoptosis (Fig. 9).

### 3.7 Effects of test fractions on the expression of the cancer-promoting gene FABP5

**Fig. 10:**
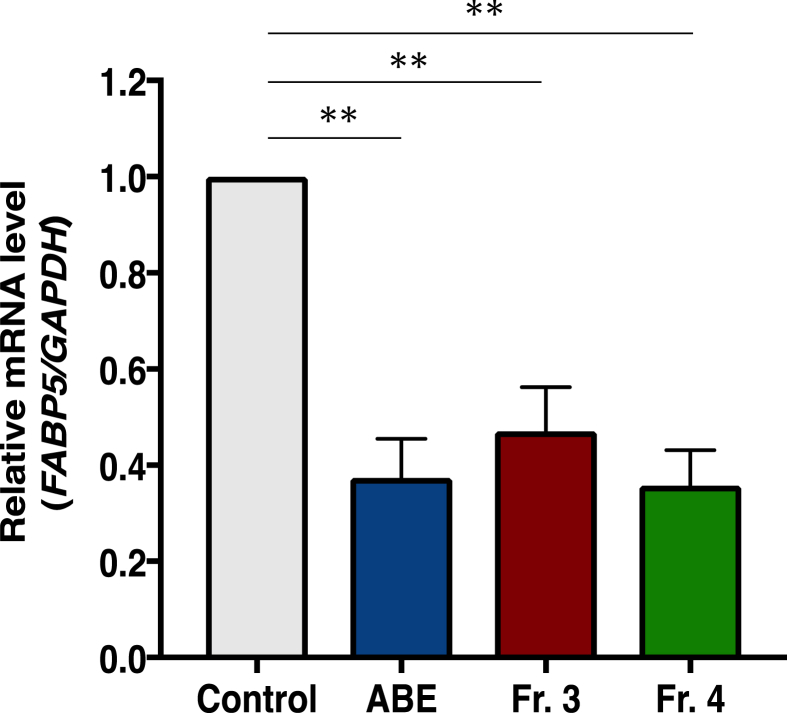
Effects of test fractions (ABE, Fr. 3, or Fr. 4) on the expression of the cancer-promoting gene *FABP5*. Test fractions (1% w/v ABE, Fr. 3, or Fr. 4) significantly suppressed the expression of the *FABP5* gene in PC-3 cells. Cells treated with these compounds for 48 h were collected and the FABP5 mRNA level was evaluated by qPCR. Relative *FABP5* expression levels were quantified by normalized levels. The data are shown as the mean ± standard deviation of three independent experiments. **P < 0.01 from one-way ANOVA followed by Dunnett's multiple comparison test.

Next, we searched for potential anti-cancer agents by assessing inhibitory activity for gene expression of the cancer-promoting gene *FABP5*. The *FABP5* gene is epigenetically regulated during human prostate carcinogenesis and is involved in growth promotion and metastasis in prostate cancer cells [12, 13, 17, 18] High-expression of *FABP5* is responsible for promotion of cell growth and invasion in various cancer cells [17, 18], suggesting that it plays a critical role in tumorigenesis of various cancer cells. The fractions derived from ABE, which suppress the expression of the *FABP5* gene, might be promising chemo-preventive agents against prostate cancer metastasis (Fig. 6). ABE, Fr.3, and Fr. 4 significantly suppressed the expression of *FABP5* at mRNA levels (Fig. 6). We have previously reported on the synthesis of epicatechin oligomers and suppression of the *FABP5* gene expression by them, and found that epicatechin pentamer significantly suppressed the expression of *FABP5* at mRNA and protein levels [11]. No suppression was observed for the units less than tetramers. Epicatechin oligomers longer than pentamers showed significant activity. Fractions 3 and 4 contained (epicatechin)_6_-catechin and (epicatechin)_7_-catechin (Fig. 2), which might be involved in suppression of expression of the *FABP5* gene (Fig. 10).

## 4 Conclusions

In conclusion, we concentrated proanthocyanidins from adzuki bean extracts by newly developed methods and characterized them. The bioactive fractions were (epicatechin)_6_-catechin and (epicatechin)_7_-catechin and their derivatives containing (epi) gallocatechins. These fractions had significant anti-cancer activity and suppressed the expression of the cancer-promoting gene *FABP5*. Our results suggest that proanthocyanidin-enriched fractions from adzuki bean extracts might be potentially chemopreventive agents for various cancers, including prostate cancer. Further in vivo and mechanistic studies are needed to confirm these anti-cancer activities.

## Declarations

### Author contribution statement

Sei-ichi Kawahara, Chisato Ishihara, Kiriko Matsumoto, Shogo Senga, Koichiro Kawaguchi, Ayaka Yamamoto: Performed the experiments; Analyzed and interpreted the data.

Jutalak Suwannachot, Yasunori Hamauzu: Analyzed and interpreted the data.

Hidefumi Makabe, Hiroshi Fujii: Conceived and designed the experiments; Analyzed and interpreted the data; Contributed reagents, materials, analysis tools or data; Wrote the paper.

### Funding statement

This work was supported in part by Chinomori Foundation, Shinshu University (to H. M. and H. F.), National Agriculture and Food Research Organization (NARO), Integration Research for Agriculture and Interdisciplinary Fields (to H. M. and H. F.), and the Tojuro Iijima Foundation for Food Science and Technology (to H. M. and H. F.).

### Competing interest statement

The authors declare no conflict of interest.

### Additional information

No additional information is available for this paper.
